# Facile Fabrication of Multilayer Chemical Filters Based on Modified Activated Carbon and Activated Carbon Fiber Nonwovens for Airborne Molecular Contaminant Removal in Semiconductor Manufacturing

**DOI:** 10.3390/polym18212774

**Published:** 2026-11-06

**Authors:** Jia-Wun Li, Chia-Lin Li, Jen-Kai Yang, Jung-Feng Chang, Yu-Heng Liao, Chih-Wei Chiu

**Affiliations:** 1 Department of Materials Science and Engineering, National Taiwan University of Science and Technology, Taipei 10607, Taiwan; 2 Origin Advanced Materials Corporation, Taipei 10557, Taiwan

**Keywords:** activated carbon, surface modification, chemical filter, activated carbon fiber, volatile organic compounds (VOCs), ammonia

## Abstract

In this study, high-performance chemical filters were developed by combining surface-modified activated carbon with activated carbon fiber (ACF) nonwoven layers to improve the removal of volatile organic compounds (VOCs) and acidic/basic gases. Activated carbon was modified using ammonium hydroxide, formic acid, and nitric acid, and the resulting pore structure, surface morphology, and functional groups were characterized using BET, FE-SEM, and XPS analyses. AC-NH_4_OH exhibited a favorable microporous structure and high specific surface area, and exhibited the best adsorption performance toward toluene, isopropyl alcohol (IPA), and acetic acid, whereas AC-HNO_3_ showed the best performance toward ammonia, possibly associated with changes in its surface functional groups. The modified activated carbon was further incorporated into chemical filters with nonwoven fabrics and adhesive. Because increasing adhesive content reduced adsorption performance through surface coverage and pore blockage, 5 wt% adhesive was selected as the preferred formulation among the adhesive-containing samples investigated. The optimized filters outperformed a commercial chemical filter for all tested gases. The incorporation of ACF provided additional microporous adsorption sites, and a single ACF layer improved the effective removal time for toluene and IPA by approximately 10.5% and 14.7%, respectively. These findings demonstrate the potential of integrating surface-modified activated carbon with ACF nonwoven layers to enhance gaseous pollutant removal for airborne molecular contaminant control in semiconductor manufacturing.

## 1. Introduction

Industrial and manufacturing processes may release volatile organic compounds (VOCs) including aromatic hydrocarbons, alcohols, and ketones. These pollutants can adversely affect human health and indoor air quality, highlighting the need for efficient air purification and filtration technologies [1,2,3,4,5,6,7]. In recent years, studies on functional polymers and composite materials have demonstrated that the final properties of materials depend not only on their composition but also strongly on filler dispersion, interfacial interactions, and microstructural design [8,9]. Through appropriate control of material composition and structure, composite materials can exhibit diverse functional properties. Such design concepts have been widely applied to various polymer nanocomposite systems [10,11,12,13] and provide useful guidance for the development of advanced functional materials [14,15,16,17,18,19].

Among the available VOC control technologies, adsorption is an efficient and economically attractive approach because it operates under relatively mild conditions, accommodates various adsorbent materials [20,21,22], and offers opportunities for adsorbent regeneration and adsorbate recovery [23,24,25]. In addition to pore structure [26], the adsorption performance of a material is also influenced by its surface chemical composition and functional groups, which may alter the interactions between adsorbate molecules and the adsorbent surface [27]. The effects of surface chemical modification on adsorption behavior have also been observed in various functionalized adsorbent systems, including the adsorption of metal ions by crosslinked β-chitosan/polyamine systems [28], the adsorption of dye molecules by crosslinked β-chitosan [29], and dye removal using β-chitosan/polyamine-functionalized graphene oxide composite adsorbents [30]. Although these systems are primarily associated with liquid-phase adsorption, their results highlight the important roles of surface functional groups, local chemical environments, and material structures in determining adsorption performance. Activated carbon (AC), owing to its high specific surface area, well-developed pore structure, and excellent adsorption capability, is one of the most widely used carbonaceous adsorbents [31,32,33]. Previous studies indicate that the adsorption behavior of VOCs on activated carbon is not governed solely by specific surface area; pore-size distribution, the fraction of micropores, and surface chemical properties are also important factors [34]. Gallego et al. evaluated the removal efficiency of indoor VOCs using coconut-shell-based activated carbon filters and demonstrated their potential for air purification applications [35]. In addition, previous reviews have indicated that the pore structure and surface functional groups of adsorbents, together with the physicochemical properties of the adsorbates, can strongly influence VOC adsorption performance [23,36]. Therefore, the simultaneous regulation of the pore structure and surface chemistry of activated carbon is an important strategy for improving its adsorption performance toward different VOCs [37,38]. Kim et al. investigated the adsorption–desorption characteristics of VOCs on impregnated activated carbon and showed that surface treatment can alter the interactions between activated carbon and gas molecules [36]. Li et al. performed surface modification of coconut-shell-based activated carbon to investigate improvements in the removal of hydrophobic VOCs [39]. Furthermore, the introduction of nitrogen-containing functional groups may modify the electronic and polar characteristics of carbon surfaces, thereby affecting VOC adsorption [40]. For basic gases, Huang et al. investigated the effect of acidic surface oxides on NH_3_ adsorption by activated carbon and identified acidic surface functional groups as one of the important factors governing ammonia adsorption [41]. These studies suggest that surface modification strategies should be tailored to the chemical characteristics of the target pollutant rather than focusing solely on maximizing individual material properties [42,43,44]. However, most studies on adsorbent modification have primarily focused on the pore structure and surface chemistry of the adsorbent itself. When granular activated carbon is further incorporated into practical air purification devices, it must be immobilized within a mechanically stable filter structure [45]. Chemical filters commonly employ nonwoven fabrics as supporting layers, with adhesives used to immobilize activated carbon particles and reduce dust generation and particle detachment during use [46,47,48]. Although adhesives facilitate filter fabrication and particle immobilization, they may also block activated carbon pores and hinder gas-phase mass transfer. Therefore, balancing particle retention and adsorption-site accessibility is essential when designing practical chemical filters. In addition to granular activated carbon, activated carbon fiber (ACF) is another widely studied carbonaceous adsorbent [49,50,51]. Suzuki systematically reviewed the fundamental characteristics and applications of ACF and identified it as a fibrous material suitable for gas adsorption and separation [52]. Subsequent studies have also shown that the pore characteristics of carbon-fiber-based adsorbents can be further tailored by adjusting the activation conditions [53]. Compared with increasing the loading of granular activated carbon, incorporating thin ACF layers into chemical filters can provide additional adsorption interfaces and may increase the number of accessible microporous adsorption sites within a limited filter thickness. However, whether the integration of ACF with surface-modified activated carbon can simultaneously improve the removal of VOCs and acidic and basic gases, and whether increasing the number of ACF layers results in proportional performance enhancement, remain to be verified through dynamic adsorption experiments.

Based on these considerations, coconut-shell-based activated carbon was employed as the primary adsorbent in this study and was separately modified using ammonium hydroxide, formic acid, and nitric acid. Field-emission scanning electron microscopy (FE-SEM), nitrogen adsorption–desorption analysis, and X-ray photoelectron spectroscopy (XPS) were used to investigate the effects of different surface modification methods on the pore structure and surface functional groups of the activated carbon. Toluene, isopropyl alcohol (IPA), acetic acid, and ammonia were subsequently used as representative gaseous pollutants to compare the adsorption behavior of the different surface-modified activated carbons. The modified activated carbons were then assembled with nonwoven fabrics and an adhesive to fabricate chemical filters, and the effect of adhesive content on filter structure and adsorption performance was evaluated. ACF layers were further incorporated into the optimized filters to investigate the removal performance of the resulting composite filters toward different types of gaseous pollutants. Through systematic comparison of material surface characteristics, filter composition, and dynamic adsorption performance, this study aims to provide useful guidance for the design of high-performance chemical filters and their practical application in gaseous pollutant control. The novelty of this study lies not in the surface modification of activated carbon alone, but in the integration of surface chemistry regulation, adhesive-content optimization, and ACF nonwoven layers into a multilayer chemical filter design. This integrated approach considers both the adsorption characteristics of modified activated carbon and the structural configuration of practical chemical filters, providing a comprehensive strategy for enhancing the removal of different gaseous pollutants.

## 2. Materials and Methods

### 2.1. Materials

Coconut-shell-based granular activated carbon (AC) was purchased from Jacobi Carbons AB (Kalmar, Sweden). Activated carbon fiber (ACF) nonwoven fabric, consisting of 50 wt% activated carbon fiber and 50 wt% polyethylene terephthalate/polypropylene (PET/PP), with a thickness of approximately 140 μm, was supplied by TOYOBO Co., Ltd. (Taipei, Taiwan). Nitric acid (HNO_3_, 65%) was purchased from Scharlau (Scharlab S.L., Sentmenat, Barcelona, Spain). Formic acid (HCOOH, 98%) was obtained from Fisher Chemical (Thermo Fisher Scientific, Waltham, MA, USA), and ammonium hydroxide solution (NH_4_OH, 28–30%) was obtained from Thermo Scientific (Thermo Fisher Scientific, Waltham, MA, USA). Polypropylene (PP) and polyethylene (PE) nonwoven fabrics used as supporting layers for chemical filter fabrication were supplied by CAI-Probonded International Co., Ltd. (Taipei, Taiwan). Toluene (99%), isopropyl alcohol (IPA, 99%), and acetic acid (CH_3_COOH, 99.8%), used as representative adsorbates, were supplied by Echo Chemical Co., Ltd. (Toufen, Miaoli, Taiwan). A mixed thermoplastic adhesive powder (K801) based on polyurethane (PU), nylon, and ethylene-vinyl acetate (EVA), with a particle size of approximately 200–300 μm, was used as the binder for chemical filter fabrication. The adhesive powder was obtained through USI Corporation (Taipei, Taiwan). Commercial activated carbon was supplied by GREENFILTEC LTD. (Taoyuan, Taiwan) and used as a reference material for adsorption performance comparison. It was designated as AC-CA. A commercially available chemical filter supplied by GREENFILTEC LTD. was used as a reference filter for performance comparison and designated as CF-CA.

### 2.2. Preparation of Surface-Modified Activated Carbon

Prior to surface modification, the pristine coconut-shell activated carbon was washed to remove residual dust and impurities. Briefly, 50 g of activated carbon was dispersed in 200 mL of deionized (DI) water and ultrasonically cleaned for 15 min at room temperature. The washing procedure was repeated until the washing solution became visually clear. The washed activated carbon was subsequently dried in a vacuum oven at 120 °C for 12 h. The resulting untreated activated carbon was denoted as AC-Pure. For surface modification, 50 g of the pre-cleaned activated carbon was placed in a 500 mL beaker and immersed in 200 mL of the corresponding modifying solution, maintaining an activated carbon-to-solution ratio of 1:4 (*w*/*v*). Ammonium hydroxide (NH_4_OH), formic acid (HCOOH), and nitric acid (HNO_3_) were separately used as modifying agents. Each suspension was continuously stirred at 800 rpm for 24 h at room temperature. After modification, the excess solution was removed, and the activated carbon was washed several times with DI water to remove residual modifying agents. The samples were then dried in a vacuum oven at 120 °C for 12 h. The resulting samples were designated as AC-NH_4_OH, AC-HCOOH, and AC-HNO_3_, respectively.

### 2.3. Fabrication of Activated-Carbon Chemical Filters

Chemical filters were fabricated using surface-modified activated carbon, thermoplastic adhesive powder, with nonwoven fabrics serving as supporting layers. Before filter fabrication, the modified activated carbon was dried in a vacuum oven at 120 °C for 2 h to remove adsorbed moisture. The dried activated carbon was mixed with thermoplastic adhesive powder at 200 rpm until a homogeneous mixture was obtained. Adhesive contents of 0, 5, 10, 25, and 50 wt% were investigated to evaluate the effects of adhesive loading on filter structure and gas adsorption performance. The adhesive content was defined as the mass ratio of adhesive powder to activated carbon, expressed as a percentage. The activated carbon/adhesive mixture was uniformly distributed between two nonwoven supporting layers to prepare 5 cm × 5 cm filter specimen. The assembled filters were subsequently placed in an oven at 60 °C for 20 min to thermally activate the adhesive powder and promote the immobilization of activated carbon particles within the nonwoven structure. Filters prepared using NH_4_OH-, HCOOH-, and HNO_3_-modified activated carbon were designated as CF-NH_4_OH, CF-HCOOH, and CF-HNO_3_, respectively. The adhesive content was indicated by a percentage suffix; for example, CF-NH_4_OH-5% denotes a chemical filter containing NH_4_OH-modified activated carbon and 5 wt% adhesive. The adhesive content was appended to the sample designation; for example, CF-NH_4_OH-5% represents a chemical filter containing NH_4_OH-modified activated carbon and 5 wt% adhesive. The sandwich-like filter structure consisted of activated carbon particles and adhesive confined between the nonwoven supporting layers.

### 2.4. Fabrication of ACF-Integrated Chemical Filters

To further enhance the adsorption performance of the chemical filters, activated carbon fiber (ACF) nonwoven layers were incorporated into the activated-carbon filter structure. The chemical filters used for ACF integration were fabricated following the procedure described in Section 2.3. Based on the adhesive-content screening, filters containing 5 wt% adhesive were selected for the subsequent ACF-layer experiments. Prior to assembly, the ACF nonwoven fabric was cut into 5 cm × 5 cm specimens and dried in a vacuum oven at 120 °C for 2 h to remove residual moisture. The dried ACF layers were then integrated with the activated-carbon chemical filter. Three filter configurations containing zero, one, or two ACF layers were prepared and designated as 0-layer ACF, 1-layer ACF, and 2-layer ACF, respectively. The ACF used in this study consisted of 50 wt% activated carbon fiber and 50 wt% PET/PP and had a thickness of approximately 140 μm. For the adsorption tests of toluene, isopropyl alcohol, and acetic acid, CF-NH_4_OH-5% was used as the base chemical filter, whereas CF-HNO_3_-5% was used for NH_3_ adsorption. The corresponding ACF-integrated samples were therefore denoted according to the number of incorporated ACF layers.

### 2.5. Characterization and Gas Adsorption Performance Evaluation

The surface morphology of the activated carbon samples and chemical filters was examined using a field-emission scanning electron microscope (FE-SEM, JEOL-6500F, JEOL Ltd., Akishima, Tokyo, Japan). Prior to FE-SEM observation, dried samples were mounted on carbon tape and coated with a thin platinum layer to improve electrical conductivity. The specific surface area and pore characteristics of the activated carbon and ACF were determined using a surface area and porosimetry analyzer (BELSORP-max, MicrotracBEL Corp., Osaka, Japan). Prior to analysis, the samples were degassed for at least 3 h to remove adsorbed moisture and gases. Nitrogen adsorption–desorption measurements were conducted at approximately 77 K using liquid nitrogen for temperature control. The specific surface area and pore characteristics were subsequently determined from the obtained adsorption–desorption isotherms. Nitrogen adsorption–desorption measurements were performed independently three times for each activated-carbon and ACF sample (*n* = 3), and the derived textural parameters were reported as mean ± standard deviation (SD). The surface chemical compositions and functional groups of the activated carbon and ACF were analyzed using X-ray photoelectron spectroscopy (XPS; PHI 5000 VersaProbe III, ULVAC-PHI, Inc., Chigasaki, Japan). Activated-carbon samples were ground into powder and dried prior to analysis. The samples were mounted on the specimen holder, and XPS measurements were performed after the chamber pressure reached approximately 2.7 × 10^−4^ Pa. The O1s and N1s spectra were analyzed to evaluate changes in oxygen- and nitrogen-containing surface functional groups induced by chemical modification. Gas adsorption performance was evaluated using a custom-built continuous-flow adsorption system equipped with mass-flow controllers (D8500, KOFLOC Corp., Kyotanabe, Japan) and a handheld gas/VOC detector (HVX501, HAL Technology, LLC, Fontana, CA, USA). All gas adsorption tests were conducted at ambient temperature. The system consisted of an air supply, two mass-flow controllers, a solvent evaporation vessel, a gas-mixing bottle, a filter-testing chamber, and a downstream gas detector. One air stream was supplied directly to the mixing chamber at 2.0 L min^−1^, whereas a second air stream at 0.2 L min^−1^ was passed through the corresponding liquid adsorbate to generate the target vapor. The two streams were subsequently mixed before being introduced into the filter-testing chamber. For VOC adsorption experiments, toluene and isopropyl alcohol (IPA) were used as representative organic vapors. The inlet concentrations were adjusted to approximately 500 ppm for toluene and 200 ppm for IPA. The NH_3_ adsorption experiment used diluted ammonia water as the gas source. 28% ammonia water and deionized water were mixed in a 1:3 ratio, and 400 mL of this solution was placed in a serum bottle. Carrier gas was introduced to generate NH_3_ vapor, and the inlet NH_3_ concentration was adjusted to approximately 130 ppm. Each 5 cm × 5 cm filter specimen was placed in the testing chamber after the inlet concentration had stabilized. Dynamic gas removal performance was evaluated based on the temporal change in removal efficiency. The time required for the removal efficiency to decrease to 80% was defined as t_80_ (min) and used as a characteristic parameter for comparing gas removal performance under the investigated experimental conditions. In addition to the characteristic removal time (t_80_), the cumulative dynamic adsorption capacity up to t_80_ (q_t80_, mg g^−1^) was calculated by integrating the removal-efficiency curves from the beginning of the adsorption experiment to the time at which the removal efficiency decreased to 80%, according to Equation (1):

(1) $$Q_{t80}=\frac{C_{in}QM}{RTm}(\int_{0}^{t80}\eta (t)dt)\times 10^{-5}$$

where C_in_ is the inlet gas concentration (ppmv), Q is the total volumetric flow rate (L min^−1^), M is the molecular weight of the target gas (g mol^−1^), R is the ideal gas constant (0.082057 L atm mol^−1^ K^−1^), T is the operating temperature (K), m is the activated-carbon mass used for normalization (g), and η(t) is the gas removal efficiency expressed as a percentage. A normalization mass of 1 g and an operating pressure of 1 atm were used for the calculation. The resulting q_t80_ values represent cumulative gas uptake up to the 80% removal-efficiency threshold rather than total saturation adsorption capacity. Pressure-drop measurements were conducted to evaluate the airflow resistance of the developed composite chemical filter. The tested sample, designated as GEACFVOC, consisted of surface-modified activated carbon, 5 wt% adhesive, and one activated carbon fiber (ACF) nonwoven layer. The pressure drop was measured at different face velocities, including 2.0, 2.5, 3.0, and 3.3 m s^−1^, and expressed in pascals (Pa). The resulting pressure-drop data were used to characterize the airflow resistance of the composite filter under different airflow conditions. Pressure-drop measurements were performed under different face-velocity conditions, and the variability of the measurements was expressed as standard deviation (SD).

### 2.6. AI-Assisted Preparation of Schematic Illustrations

**Figure 1:**
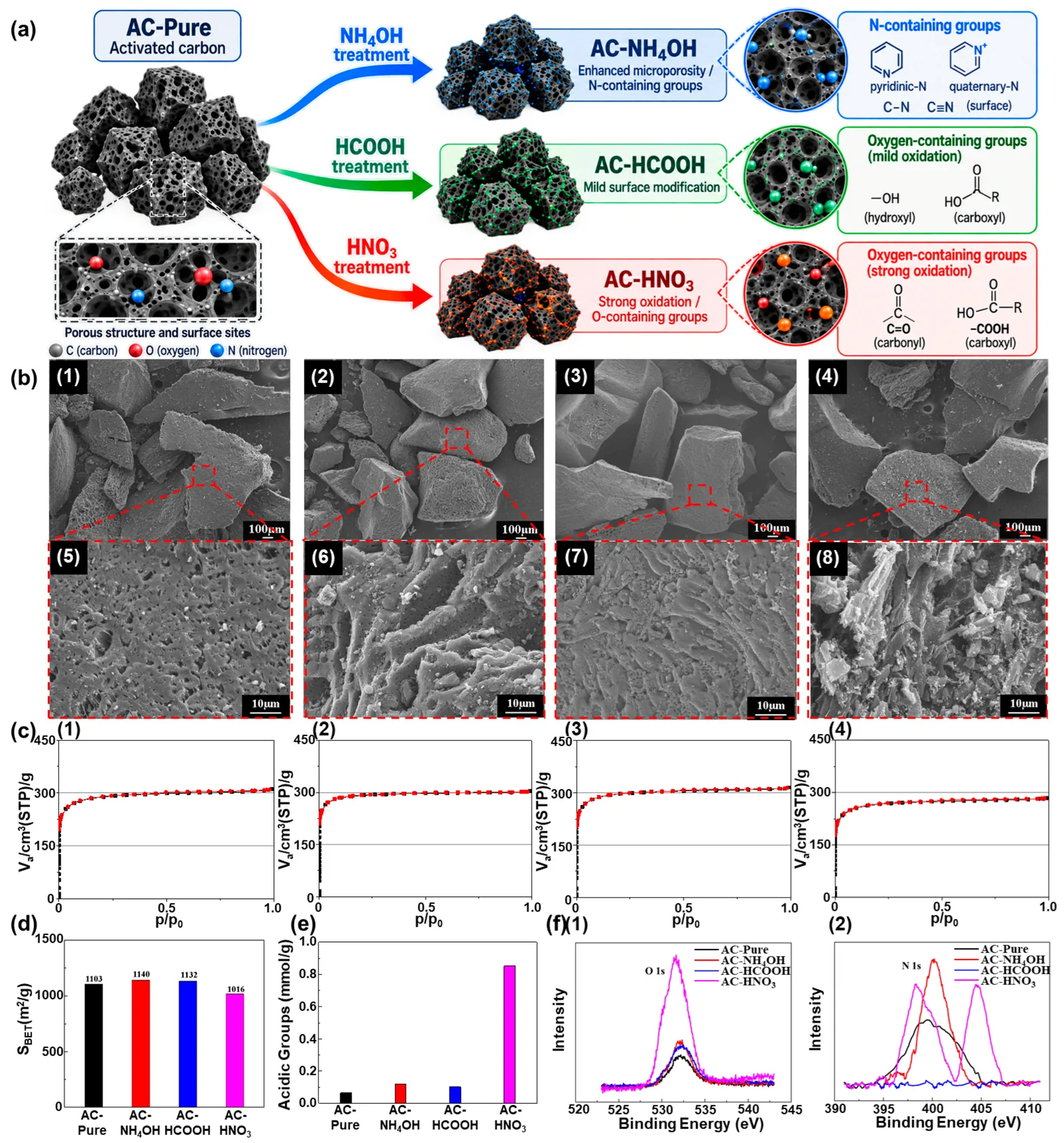
Effects of different chemical modification methods on the structure and surface chemistry of activated carbon. (**a**) Schematic illustration of activated-carbon surface modification using NH_4_OH, HCOOH, and HNO_3_ solutions, with treatment performed under stirring at room temperature for 24 h; the functional-group representations illustrate possible surface chemical changes rather than directly identified reaction products; (**b**) FE-SEM images of different activated carbons: (**1**–**4**) correspond to AC-Pure, AC-NH_4_OH, AC-HCOOH, and AC-HNO_3_, respectively, and (**5**–**8**) show the corresponding higher-magnification images; (**c**) nitrogen adsorption–desorption isotherms of (**1**) AC-Pure, (**2**) AC-NH_4_OH, (**3**) AC-HCOOH, and (**4**) AC-HNO_3_, where the red and black curves represent the adsorption and desorption branches, respectively; (**d**) BET specific surface area; (**e**) content of acidic surface functional groups; and (**f**) XPS spectra of (**1**) O 1s and (**2**) N 1s. (**a**) presents a schematic interpretation of the proposed effects of surface modification and was prepared with the assistance of ChatGPT (OpenAI) for conceptual visualization. Panels (**b**–**f**) present experimentally obtained characterization results.

**Figure 2:**
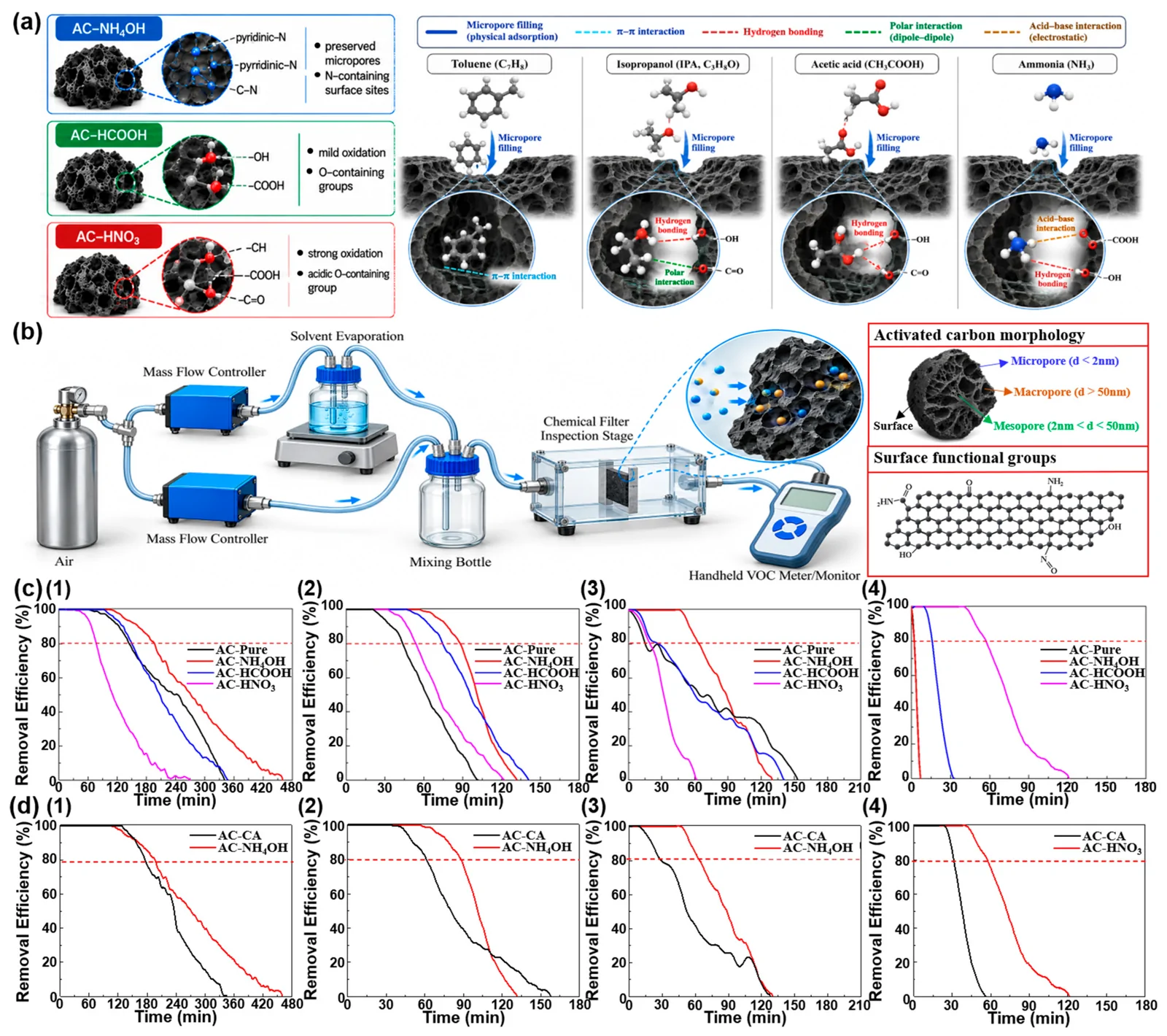
Possible adsorption mechanisms, dynamic gas removal performance of surface-modified activated carbons, and comparison with commercial activated carbon. (**a**) Schematic illustration of the surface characteristics and possible adsorption interactions of the differently modified activated carbons. (**b**) Schematic representation of the continuous-flow gas adsorption testing system used to evaluate the activated-carbon samples, together with conceptual illustrations of their pore structures and surface functional groups; the depicted pore structures and functional groups are illustrative and are not direct microscopic observations; (**c**) dynamic gas removal performance of AC-Pure, AC-NH_4_OH, AC-HCOOH, and AC-HNO_3_; and (**d**) performance comparison between the selected modified activated carbons and commercial activated carbon (AC-CA). In (**c**) and (**d**), (**1**–**4**) correspond to toluene, isopropyl alcohol (IPA), acetic acid (CH_3_COOH), and ammonia (NH_3_), respectively. AC-NH_4_OH was used for comparison with the commercial activated carbon for toluene, IPA, and CH_3_COOH, whereas AC-HNO_3_ was used for NH_3_. The red dashed line indicates a removal efficiency of 80%. The schematic illustrations in (**a**,**b**) were prepared with the assistance of ChatGPT (OpenAI) for conceptual visualization and represent proposed adsorption mechanisms and the experimental configuration, rather than direct experimental observations. The dynamic gas removal data in (**c**,**d**) were obtained experimentally.

**Figure 3:**
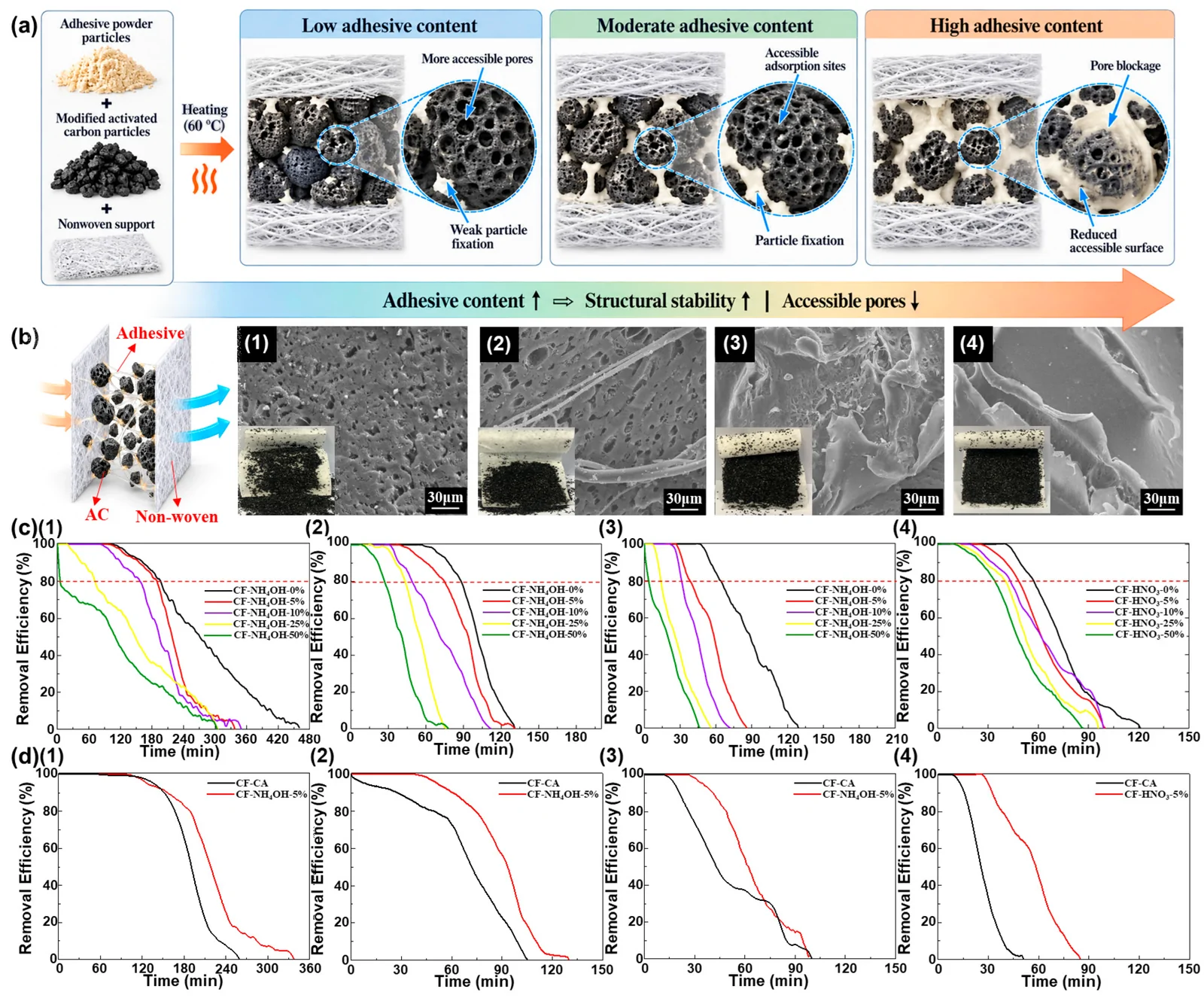
Effects of adhesive content on the structure and dynamic gas removal performance of chemical filters. (**a**) Conceptual schematic illustrating the proposed influence of increasing adhesive content on activated-carbon particle immobilization, accessible adsorption sites, and potential pore blockage; the illustrated changes in particle fixation and structural stability are qualitative interpretations rather than results of direct mechanical testing; (**b**) schematic structure of the activated carbon/adhesive/nonwoven composite filter and FE-SEM images at different adhesive contents: (**1**) 5 wt%, (**2**) 10 wt%, (**3**) 25 wt%, and (**4**) 50 wt%. The inset in each image shows the corresponding macroscopic appearance of the filter; (**c**) dynamic gas removal performance of filters containing 0, 5, 10, 25, and 50 wt% adhesive; and (**d**) performance comparison between the filter containing 5 wt% adhesive and the commercial chemical filter (CF-CA). In (**c**) and (**d**), (**1**–**4**) correspond to toluene, IPA, CH_3_COOH, and NH_3_, respectively. Filters containing NH_4_OH-modified activated carbon were used for the toluene, IPA, and CH_3_COOH tests, whereas filters containing HNO_3_-modified activated carbon were used for the NH_3_ test. The red dashed line indicates a removal efficiency of 80%. The schematic illustrations in (**a**) and the schematic components in (**b**) were prepared with the assistance of ChatGPT (OpenAI) to illustrate the proposed effects of adhesive content and filter structure. The FE-SEM images and macroscopic photographs in (**b**), together with the dynamic gas removal data in (**c**,**d**), were obtained experimentally.

**Figure 4:**
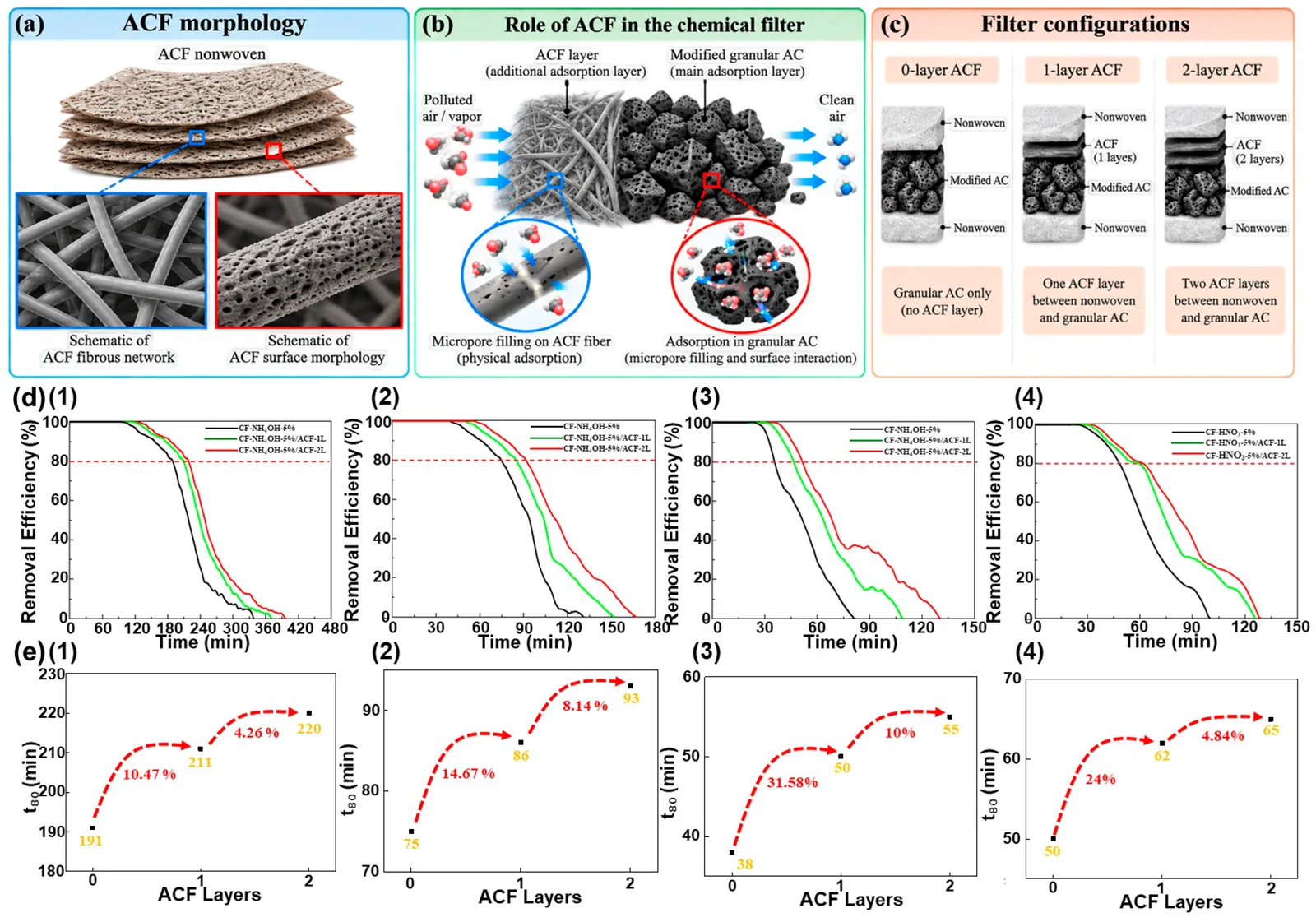
Effect of integrating activated carbon fiber (ACF) nonwoven layers on the dynamic gas removal performance of chemical filters. (**a**) Schematic illustrations of the ACF nonwoven structure, fibrous network, and surface morphology; (**b**) schematic illustration of the possible adsorption contributions of the ACF layers and modified granular activated carbon in the composite filter; (**c**) filter configurations containing 0, 1, and 2 ACF layers; (**d**) dynamic gas removal performance of filters containing different numbers of ACF layers; and (**e**) t_80_ values at different numbers of ACF layers and the relative improvements between successive ACF-layer configurations. In (**d**,**e**), (**1**–**4**) correspond to toluene, IPA, CH_3_COOH, and NH_3_, respectively. CF-NH_4_OH-5% was used as the base filter for the toluene, IPA, and CH_3_COOH tests, whereas CF-HNO_3_-5% was used as the base filter for the NH_3_ test. t_80_ is defined as the time required for the removal efficiency to decrease to 80%. The red dashed line in (**d**) indicates a removal efficiency of 80%. In (**e**), the yellow numbers indicate the t_80_ values (min) for each ACF-layer configuration, whereas the red numbers and dashed arrows indicate the relative percentage improvements in t_80_ between successive ACF-layer configurations (0 to 1 layer and 1 to 2 layers). The illustrations in (**a**–**c**) were prepared with the assistance of ChatGPT (OpenAI) for conceptual visualization. The dynamic adsorption results in (**d**,**e**) were obtained experimentally.

The graphical abstract and conceptual schematic illustrations in Figure 1a, Figure 2a,b, Figure 3a,b and Figure 4a–c were prepared with the assistance of ChatGPT (GPT-5.6 Sol, OpenAI, San Francisco, CA, USA) in October 2026 for scientific visualization purposes. The illustrations were developed through an iterative process involving initial image generation and subsequent refinements to scientific labels, chemical structures, colors, layouts, and visual presentation. The principal prompting instructions concerned the surface modification of activated carbon using NH_4_OH, HCOOH, and HNO_3_; possible adsorption interactions involving toluene, IPA, CH_3_COOH, and NH_3_; the influence of adhesive content on particle immobilization and accessible adsorption sites; and the conceptual role of ACF layers in composite chemical filters. These descriptions summarize the main themes of the prompting process rather than verbatim individual prompts. The AI-assisted illustrations were used solely for conceptual visualization and do not constitute direct experimental observations. Experimentally obtained FE-SEM images, BET and XPS characterization results, and dynamic gas adsorption data were distinguished from the schematic illustrations and were not generated or modified using AI tools. The authors reviewed the illustrations for scientific accuracy and take responsibility for their final presentation.

## 3. Results and Discussion

### 3.1. Structural and Surface Characteristics of Surface-Modified Activated Carbon

The effects of different chemical modification methods on the pore structure, surface morphology, and surface chemical properties of activated carbon were first investigated. Coconut-shell-based activated carbon was separately modified using ammonium hydroxide, formic acid, and nitric acid. The pristine and modified activated carbons were designated as AC-Pure, AC-NH_4_OH, AC-HCOOH, and AC-HNO_3_, respectively, and the corresponding characterization results are presented in Figure 1. Figure 1a schematically illustrates the possible changes in the pore structure and surface functional groups of activated carbon after treatment with NH_4_OH, HCOOH, and HNO_3_. NH_4_OH modification was primarily beneficial for preserving and developing the microporous structure while introducing nitrogen-containing functional groups. HCOOH treatment exerted a relatively mild effect on the activated-carbon structure. In contrast, HNO_3_ exhibited stronger oxidative activity, which could increase the abundance of oxygen-containing and acidic surface functional groups, but might simultaneously induce partial pore blockage or erosion of the carbon framework. The surface morphologies of the activated carbons before and after modification are shown in Figure 1(b1–4), while Figure 1(b5–8) presents the corresponding higher-magnification FE-SEM images. Figure 1(b1,5) shows AC-Pure, which exhibited an irregular particulate morphology and a porous surface structure characteristic of the pristine coconut-shell-based activated carbon. Figure 1(b2,6) shows AC-NH_4_OH, for which a greater number of relatively small pores were observed on the surface, indicating that a well-developed porous structure was retained after NH_4_OH modification. Figure 1(b3,7) shows AC-HCOOH, whose surface morphology remained similar to that of AC-Pure, although changes in surface structure induced by chemical modification could still be observed. Figure 1(b4,8) shows AC-HNO_3_, which exhibited more pronounced surface erosion and localized fragmented regions, suggesting that the strong oxidative treatment exerted a greater influence on the carbon framework. The FE-SEM observations were consistent with the pore-structure analysis: AC-NH_4_OH exhibiting a greater number of small surface pores, whereas AC-HNO_3_ showed more pronounced fragmentation and morphological changes. The nitrogen adsorption–desorption isotherms of the different activated carbons are shown in Figure 1(c1–4), corresponding to AC-Pure, AC-NH_4_OH, AC-HCOOH, and AC-HNO_3_, respectively. All samples exhibited pronounced nitrogen uptake in the low-relative-pressure region, indicating that the pore structures of the four activated carbons were dominated by micropores. Detailed pore-structure parameters are summarized in Table S1. Micropores play an important role in gas adsorption because stronger adsorbent–adsorbate interactions within narrow pores promote pore filling at low relative pressures. The differences induced by the various chemical modification methods can be further observed from the BET specific surface areas shown in Figure 1d. The specific surface areas of AC-Pure, AC-NH_4_OH, AC-HCOOH, and AC-HNO_3_ were 1101 ± 7, 1142 ± 10, 1134 ± 8, and 1018 ± 11 m^2^ g^−1^, respectively (mean ± SD, *n* = 3). Among the four samples, AC-NH_4_OH exhibited the highest specific surface area, while AC-HCOOH also showed a slight increase after modification. In contrast, the specific surface area of AC-HNO_3_ decreased markedly. This decrease may be associated with the stronger oxidative and acidic nature of nitric acid, which, while increasing the amount of oxidized surface functional groups, may also cause partial pore blockage and erosion of the carbon framework. Overall, NH_4_OH modification resulted in the highest mean BET specific surface area and micropore volume fraction among the investigated samples, which may provide a greater number of accessible sites for gas adsorption. In addition to modifying the pore structure, chemical treatment also altered the acid–base properties of the activated-carbon surface. As shown in Figure 1e, AC-HNO_3_ exhibited a substantially higher content of acidic functional groups than AC-Pure, AC-NH_4_OH, and AC-HCOOH. This result indicates that the strong oxidative effect of HNO_3_ promotes the formation of acidic oxygen-containing functional groups on the activated-carbon surface. The enhanced surface acidity introduced by HNO_3_ modification was also associated with the subsequently improved NH_3_ adsorption performance. To further evaluate the surface chemical composition after modification, XPS was employed to analyze changes in oxygen- and nitrogen-containing functional groups. The XPS spectra provide further information on the surface chemical environments of the modified activated carbons. As shown in Figure 1(f1), the O 1s spectra exhibit broad features in the approximately 531–533 eV binding-energy region, consistent with oxygen-containing surface species. However, because different oxygen functional groups may contribute to overlapping signals within this region, their individual contributions cannot be quantitatively resolved from the spectra presented. As shown in Figure 1(f2), the N 1s spectra reveal differences in nitrogen-related surface chemical environments following the modification treatments. AC-NH_4_OH exhibits a prominent peak near 400 eV, which may be associated with pyrrolic-N- or amine-related species. In contrast, AC-HNO_3_ exhibits two characteristic features at approximately 398–399 eV and 404–405 eV. Based on commonly reported XPS binding-energy assignments, these features may be tentatively associated with pyridinic-N-related and oxidized nitrogen species, respectively. The higher-binding-energy feature may also include contributions from other highly oxidized nitrogen species associated with nitric acid treatment. These observations suggest differences in nitrogen-related surface chemistry between NH_4_OH- and HNO_3_-modified activated carbons. However, because quantitative peak deconvolution was not performed, the relative proportions and precise chemical identities of these nitrogen species cannot be conclusively determined. The BET results showed that AC-NH_4_OH had the highest specific surface area (1142 ± 10 m^2^ g^−1^), whereas AC-HNO_3_ had the lowest (1018 ± 11 m^2^ g^−1^) among the investigated samples. Nevertheless, AC-HNO_3_ exhibited the best NH_3_ removal performance, indicating that specific surface area alone does not explain the observed adsorption behavior. Together with the greater abundance of acidic surface functional groups observed for AC-HNO_3_, the combined characterization results suggest that changes in surface chemistry may contribute to differences in gas removal performance. However, the relative contributions of pore structure and specific surface functional groups cannot be quantitatively separated using the available characterization and dynamic adsorption data. Although the combined BET, FE-SEM, XPS, and surface acidity analyses provide information on the pore structure, surface morphology, and chemical environments of the modified activated carbons, changes in carbon-framework disorder and the spatial distribution of surface elements were not directly characterized. Raman spectroscopy and elemental mapping would provide complementary information regarding these aspects and are therefore considered valuable directions for further investigation.

### 3.2. Gas Adsorption Performance of Surface-Modified Activated Carbon

To elucidate the differences in gas adsorption performance among the surface-modified activated carbons, Figure 2a summarizes the possible pore-filling and surface interactions involved in the adsorption process. Toluene adsorption may involve pore filling as well as π–π interactions between its aromatic ring and the carbon surface. In addition to pore filling, isopropyl alcohol (IPA) and acetic acid (CH_3_COOH) may interact with polar surface functional groups through hydrogen bonding and other intermolecular interactions. For ammonia (NH_3_), the higher surface acidity resulting from nitric acid treatment may favor adsorption. The interactions illustrated above represent possible adsorption mechanisms proposed on the basis of the material characterization and adsorption results; however, the individual contributions of these interactions to the overall adsorption performance require further verification. To evaluate the gas removal performance of the different surface-modified activated carbons, adsorption tests were conducted using the custom-built continuous-flow gas adsorption system shown in Figure 2b and Video S1, with toluene, IPA, CH_3_COOH, and NH_3_ selected as the target gases. Adsorption performance was primarily compared using t_80_, defined as the time required for the gas removal efficiency to decrease to 80%. However, t_80_ characterizes the effective gas removal duration and does not directly quantify the amount of gas adsorbed per unit mass of activated carbon. Under identical test conditions, a longer t_80_ indicates that the sample can maintain a removal efficiency of at least 80% for a longer period. The toluene removal profiles of the different surface-modified activated carbons are shown in Figure 2(c1), and the corresponding data are summarized in Table 1. Among the four activated carbons, AC-NH_4_OH exhibited the longest t_80_ of 197 min, indicating that NH_4_OH modification was beneficial for improving the dynamic removal performance toward toluene. The IPA removal profiles are presented in Figure 2(c2), and the corresponding t_80_ values are listed in Table 1. AC-NH_4_OH again exhibited the best performance, with a t_80_ of 88 min. These results are consistent with the trends observed in the preceding pore-structure analysis. AC-NH_4_OH exhibited a higher specific surface area and micropore volume fraction, providing a pore structure favorable for gas adsorption. In addition, changes in surface chemistry may alter the interactions between the activated-carbon surface and gas molecules, thereby contributing to the overall removal performance. The acetic acid removal profiles are shown in Figure 2(c3), and the corresponding data are summarized in Table 1. Similar to the results for toluene and IPA, AC-NH_4_OH exhibited the best dynamic removal performance toward acetic acid, with a t_80_ of 64 min. This behavior may be associated with its higher micropore volume fraction and the surface chemical properties introduced by modification. Micropores provide adsorption space, while polar surface functional groups may interact with acetic acid molecules through hydrogen bonding and other intermolecular interactions, thereby prolonging the effective removal time. A different trend was observed for NH_3_. As shown in Figure 2(c4) and summarized in Table 1, AC-HNO_3_ exhibited the best NH_3_ removal performance, with a t_80_ of 57 min, despite its relatively lower specific surface area. This result indicates that NH_3_ removal is not governed solely by specific surface area and that the surface chemistry of activated carbon also plays an important role. The higher surface acidity introduced by HNO_3_ modification may promote acid–base interactions with basic NH_3_ molecules. In addition, oxygen-containing surface functional groups may participate in hydrogen-bonding interactions, thereby contributing to the enhanced NH_3_ removal performance. To further evaluate the performance of the modified activated carbons relative to commercial activated carbon, the modified sample exhibiting superior performance toward each target gas was compared with commercial activated carbon (AC-CA), as shown in Figure 2d, with the corresponding data summarized in Table 1. For toluene, as shown in Figure 2(d1), AC-NH_4_OH exhibited a t_80_ of 197 min, which was 22 min longer than the 175 min obtained for AC-CA. For IPA, Figure 2(d2) shows that AC-NH_4_OH exhibited a t_80_ of 88 min, representing an extension of 27 min relative to the 61 min observed for AC-CA. For acetic acid, as shown in Figure 2(d3), AC-NH_4_OH exhibited a t_80_ of 64 min, which was 35 min longer than the 29 min obtained for AC-CA. For NH_3_, AC-HNO_3_ exhibited a t_80_ of 57 min, which was 25 min longer than that of AC-CA (32 min), as shown in Figure 2(d4) These results demonstrate that, under the test conditions employed in this study, selecting an appropriate surface modification according to the target gas can prolong the period during which activated carbon maintains an effective removal efficiency. The enhancement achieved by chemical modification varied with the type of gaseous pollutant. The pore structure and surface properties produced by NH_4_OH modification were favorable for the removal of toluene, IPA, and acetic acid, whereas HNO_3_ modification was associated with higher surface acidity and improved NH_3_ removal performance. These findings indicate that the design of surface-modified activated carbon should simultaneously consider pore structure, surface chemistry, and the physicochemical characteristics of the target gas to improve dynamic gas removal performance. From the perspective of adsorption mechanisms, the gas removal behavior of surface-modified activated carbon can be interpreted in terms of physical adsorption and surface chemical interactions. Physical adsorption is mainly associated with pore filling within the microporous structure of activated carbon, where the accessible surface area and pore characteristics influence the adsorption of gas molecules. In this study, AC-NH_4_OH exhibited the highest specific surface area (1142 m^2^ g^−1^) and micropore volume fraction (96.84 ± 2.35%), which may explain its favorable adsorption performance toward toluene, IPA, and acetic acid. Compared with AC-Pure, AC-NH_4_OH exhibited an increase in specific surface area from 1101 to 1142 m^2^ g^−1^ (approximately 3.7%) and in micropore volume fraction from 94.53% to 96.84%, accompanied by an increase in the toluene t_80_ value from 144 to 197 min (approximately 36.8%). The substantially greater relative improvement in dynamic removal time compared with BET specific surface area suggests that surface area alone cannot fully explain the observed adsorption performance. Changes in pore characteristics and surface chemical properties may both contribute to the enhanced VOC removal behavior, although their individual contributions cannot be quantitatively distinguished from the present data. In addition, π–π interactions between aromatic molecules and the carbon surface may contribute to toluene adsorption, whereas hydrogen bonding and other intermolecular interactions involving polar surface functional groups may influence IPA and acetic acid adsorption. In contrast, AC-HNO_3_ exhibited a lower specific surface area (1018 m^2^ g^−1^) but showed the highest NH_3_ removal performance (t_80_ = 57 min). Combined with the XPS and surface acidity results, this observation suggests that surface chemical properties may play a more significant role in NH_3_ adsorption. The increased abundance of acidic and oxygen-containing surface functional groups following HNO_3_ treatment may promote acid–base interactions with NH_3_ molecules, thereby enhancing their removal. Overall, these findings suggest that both pore structure and surface chemistry contribute to gas adsorption, with their relative importance depending on the physicochemical characteristics of the target gas. Nevertheless, the present results do not allow the individual contributions of physical adsorption and surface chemical interactions to be quantitatively distinguished. Therefore, the proposed adsorption mechanisms should be regarded as interpretations supported by the existing characterization and adsorption results rather than independently verified mechanistic pathways. To complement the t_80_-based comparison, the cumulative dynamic adsorption capacities up to t_80_ (q_t80_, mg g^−1^) were calculated by integrating the corresponding gas removal-efficiency curves using Equation (1). The adsorption capacities were normalized to the experimentally confirmed activated-carbon mass of 1 g, and the results are summarized in Table S2 in the Supplementary Materials. Unlike t_80_, which represents the effective gas removal duration, q_t80_ provides a mass-normalized measure of the cumulative gas uptake up to the 80% removal-efficiency threshold. These two parameters therefore provide complementary information on dynamic adsorption performance, and the reported q_t80_ values should not be interpreted as total saturation adsorption capacities.

### 3.3. Effect of Adhesive Content on the Adsorption Performance of Chemical Filters

To incorporate the surface-modified activated carbon into chemical filters, nonwoven fabrics were used as supporting layers, and an adhesive was introduced to immobilize the activated carbon particles. Figure 3a schematically illustrates the effects of adhesive content on particle immobilization and surface coverage. A low adhesive content preserves a larger fraction of the exposed activated-carbon surface but may provide insufficient particle fixation. In contrast, increasing the adhesive content improves particle immobilization but may also increase surface coverage and block pore entrances. Therefore, the filter morphology and gas removal performance at different adhesive contents were compared to evaluate the influence of adhesive loading on filter design. Figure 3b presents the structure and FE-SEM images of the activated carbon/adhesive/nonwoven composite filters. The insets show the corresponding macroscopic appearance of each filter. Figure 3(b1) shows the sample containing 5 wt% adhesive, in which a relatively large number of visible pores remained exposed on the activated-carbon surface, indicating a comparatively low degree of surface coverage. Figure 3(b2) and Figure 3(b3) correspond to adhesive contents of 10 and 25 wt%, respectively. As the adhesive content increased, progressively greater coverage of the activated-carbon surface was observed. At an adhesive content of 50 wt%, as shown in Figure 3(b4), the activated-carbon surface exhibited substantially more pronounced coverage. The macroscopic filter appearance also indicated poorer particle retention at lower adhesive contents, whereas increasing the adhesive content improved immobilization of the activated carbon on the nonwoven support. These observations indicate that although increasing the adhesive content is beneficial for particle fixation, it may simultaneously reduce the adsorption surface accessible to gas molecules. The effect of adhesive content on toluene removal performance is shown in Figure 3(c1), and the corresponding data are summarized in Table 2. Because the preceding results showed that AC-NH_4_OH exhibited favorable toluene removal performance, this modified activated carbon was used to prepare filters containing 0, 5, 10, 25, and 50 wt% adhesive. The sample without adhesive exhibited the longest effective removal time. After adhesive addition, the time required for the removal efficiency to decrease to 80% (t_80_) became shorter. Among the adhesive-containing formulations, the 5 wt% sample retained the best toluene removal performance, whereas further increases in adhesive content led to more pronounced performance deterioration. This trend is consistent with the surface coverage observed by FE-SEM, suggesting that adhesive loading affects the accessibility of the adsorption surface. The IPA removal results are shown in Figure 3(c2) and summarized in Table 2. AC-NH_4_OH was likewise used to prepare filters with different adhesive contents for these experiments. Similar to the results for toluene, the addition of adhesive decreased the t_80_ for IPA, with the 5 wt% formulation showing the smallest reduction. As the adhesive content increased further, the period during which the filter maintained a high removal efficiency became progressively shorter. These results suggest that surface coverage caused by the adhesive may adversely affect the dynamic removal performance toward both toluene and IPA. The acetic acid removal results are presented in Figure 3(c3) and summarized in Table 2. Based on the preceding screening results for the modified activated carbons, AC-NH_4_OH was also employed as the adsorbent in this set of experiments. Overall, t_80_ for acetic acid decreased with increasing adhesive content, while the 5 wt% formulation retained the best removal performance among the adhesive-containing samples. The decline in performance at higher adhesive contents may be attributed to coverage of the activated-carbon surface and pore openings, which reduces the number of accessible adsorption sites. The NH_3_ removal results are shown in Figure 3(c4) and summarized in Table 2. Because AC-HNO_3_ exhibited the best NH_3_ removal performance in the preceding experiments, this modified activated carbon was selected for preparing filters with different adhesive contents. The addition of adhesive likewise shortened the effective NH_3_ removal time, while the 5 wt% formulation showed the smallest degree of performance loss. These results indicate that reduced accessibility of the adsorption surface caused by the adhesive may also limit the NH_3_ removal performance of the AC-HNO_3_-based filter. To compare the effects of adhesive loading on all four target gases, the t_80_ values of the different formulations are summarized in Figure S1 in the Supplementary Materials. For all four target gases, the effective removal times decreased with increasing adhesive content. Among the adhesive-containing formulations evaluated in this study, the 5 wt% sample exhibited the smallest adverse effect on gas removal performance and was therefore selected as the base formulation for subsequent comparison with the commercial filter and for ACF integration experiments. The selection of 5 wt% adhesive was based on the combined consideration of the FE-SEM observations and dynamic gas adsorption results. At this adhesive content, the activated carbon particles could be incorporated into the nonwoven filter structure while maintaining relatively exposed carbon surfaces and accessible pores. In contrast, increasing the adhesive content to 10–50 wt% resulted in progressively greater surface coverage, potentially blocking pore entrances and reducing the available adsorption sites. These morphological observations were consistent with the decreasing t_80_ values observed for the investigated gases as the adhesive content increased. Therefore, 5 wt% was selected as the most suitable formulation among the adhesive-containing samples evaluated in this study, rather than as a universally optimized adhesive content. Nevertheless, the morphological and macroscopic observations provide only qualitative information regarding particle immobilization and filter integrity. Quantitative measurements of particle shedding, adhesion strength, and mechanical durability were not performed. Further investigations are therefore required to establish the long-term structural stability of the proposed chemical filters under practical operating conditions. Quantitative measurements of particle shedding, adhesion strength, and mechanical durability were not performed. Further investigations are therefore required to establish the long-term structural stability of the proposed chemical filters under practical operating conditions. The selected chemical filters were further compared with the commercial chemical filter (CF-CA), as shown in Figure 3d, with the corresponding data summarized in Table 2. For toluene, as shown in Figure 3(d1), CF-NH_4_OH-5% exhibited a t_80_ of 190 min, which was 23 min longer than the 167 min obtained for the commercial filter, corresponding to an improvement of approximately 13.8%. For IPA, Figure 3(d2) shows that CF-NH_4_OH-5% exhibited a t_80_ of 75 min, which was 23 min longer than the 52 min obtained for the commercial filter, corresponding to an improvement of approximately 44.2%. For acetic acid, as shown in Figure 3(d3), CF-NH_4_OH-5% exhibited a t_80_ of 38 min, which was twice that of the commercial filter (19 min). For NH_3_, as shown in Figure 3(d4), CF-HNO_3_-5% exhibited a t_80_ of 49 min, which was 23 min longer than the 26 min obtained for the commercial filter, corresponding to an improvement of approximately 88.5%. These results demonstrate that, under the test conditions employed in this study, the selected modified-activated-carbon filters exhibited longer effective removal times for all four target gases than the commercial filter. The gas removal performance of the chemical filters was influenced not only by the pore structure and surface chemistry of the activated carbon but also by the adhesive content. Increasing the adhesive content improved particle immobilization, but excessive adhesive loading increased surface coverage and reduced the accessibility of adsorption sites. Among the adhesive-containing formulations, the 5 wt% samples retained the best dynamic removal performance. Accordingly, CF-NH_4_OH-5% and CF-HNO_3_-5% exhibited higher t_80_ values than the commercial filter for their respective target gases and were selected as the base filters for subsequent integration of ACF layers.

### 3.4. Enhancement of Chemical Filter Adsorption Performance Through Integration of Activated Carbon Fiber Layers

Before integrating ACF into the chemical filters, the pore structure of the ACF nonwoven fabric was first characterized. Figure S2 in the Supplementary Materials shows the nitrogen adsorption–desorption isotherm of the ACF, and the corresponding pore-structure parameters are summarized in Table S3 in the Supplementary Materials. The ACF exhibited a BET specific surface area of 512.33 ± 39.01 m^2^ g^−1^ and a micropore volume fraction of 98.91 ± 0.57% (mean ± SD, *n* = 3), indicating that its pore structure was predominantly microporous. Although the specific surface area of the ACF was lower than that of the granular activated carbon used in this study, its incorporation into the original chemical filter provided additional adsorptive material and gas-contact interfaces, thereby further improving the overall gas removal performance of the filter. The ACF nonwoven fabric used in this study consisted of 50 wt% activated carbon fiber and 50 wt% PET/PP, with an overall thickness of approximately 140 μm. Schematic illustrations of the ACF nonwoven structure, fibrous network, and surface morphology are presented in Figure 4a. These illustrations are intended for conceptual visualization and do not represent experimentally acquired FE-SEM images. Figure 4b schematically illustrates the possible adsorption contributions of the ACF layers and modified granular activated carbon within the composite filter. The ACF layers provide additional adsorption interfaces and participate in gas removal together with the original granular activated carbon. The filter configurations containing zero, one, and two ACF layers are illustrated in Figure 4c. Based on the preceding screening results, CF-NH_4_OH-5% was used as the base filter for the toluene, IPA, and acetic acid tests, whereas CF-HNO_3_-5% was used for the NH_3_ test. The dynamic removal profiles of the filters containing different numbers of ACF layers are shown in Figure 4d. The corresponding times required for the removal efficiency to decrease to 80% (t_80_) and the associated performance improvements are summarized in Figure 4e and Table 2. The dynamic removal results for toluene are shown in Figure 4(d1). After the addition of ACF, the period during which the filter maintained a high removal efficiency was extended. As shown in Figure 4(e1), the t_80_ value was 191 min without ACF, increased to 211 min after the addition of one ACF layer, corresponding to an improvement of approximately 10.47%, and further increased to 220 min with two ACF layers. Relative to the one-layer configuration, the second ACF layer provided an additional improvement of approximately 4.26%. These results indicate that ACF integration prolonged the effective removal time for toluene, although the additional benefit of second layer was smaller than of the first layer. A similar trend was observed for IPA, as shown in Figure 4(d2). According to Figure 4(e2), the t_80_ value increased from 75 min without ACF to 86 min with one ACF layer, corresponding to an improvement of approximately 14.67%. With two ACF layers, t_80_ further increased to 93 min, representing an additional improvement of approximately 8.14% relative to the one-layer configuration. As observed for toluene, the first ACF layer produced a greater improvement in IPA removal performance than second layer. The dynamic removal results for acetic acid are shown in Figure 4(d3). As shown in Figure 4(e3), the t_80_ value increased from 38 min without ACF to 50 min with one ACF layer, corresponding to an improvement of approximately 31.58%. After the addition of a second ACF layer, t_80_ further increased to 55 min, representing an additional improvement of 10% relative to the one-layer configuration. Based on the relative improvement achieved by the first ACF layer, acetic acid exhibited the largest enhancement among the four tested gases, indicating that the introduction of ACF was particularly effective in prolonging the effective removal time for acetic acid. The dynamic removal results for NH_3_ are shown in Figure 4(d4). According to Figure 4(e4), the t_80_ value increased from 50 min without ACF to 62 min with one ACF layer, corresponding to a 24% improvement. After the addition of a second ACF layer, t_80_ further increased to 65 min, representing an additional improvement of approximately 4.84% relative to the one-layer configuration. These results indicate that incorporating ACF layers into CF-HNO_3_-5% also prolonged the effective NH_3_ removal time, although the additional contribution of the second layer was comparatively limited. The performance enhancement observed after ACF integration may be associated with its highly microporous structure, which provides additional adsorption sites and increases the number of accessible adsorption interfaces beyond those supplied by the granular activated carbon. However, because the addition of ACF also increased the total amount of adsorptive material in the filter, the present results primarily reflect an improvement in the overall removal performance of the composite filter. Therefore, these results alone do not provide sufficient evidence to establish a synergistic effect between ACF and granular activated carbon. Overall, the addition of one ACF layer increased the t_80_ values for toluene, IPA, acetic acid, and NH_3_ by approximately 10.47%, 14.67%, 31.58%, and 24%, respectively. Increasing the number of ACF layers from one to two provided further improvements for all four gases; however, both the absolute increases in effective removal time and the relative gains were smaller than those obtained with the first ACF layer. This indicates that, within the range investigated in this study, increasing the number of ACF layers did not result in proportional performance enhancement. In addition to the t_80_-based comparison, the cumulative gas uptake up to t_80_ was calculated for the composite filters containing zero, one, and two ACF layers, as summarized in Table S4 of the Supplementary Materials. For toluene, the cumulative gas uptake increased from 753.0 mg without ACF to 839.1 and 876.4 mg with one and two ACF layers, respectively. Similar trends were observed for IPA (77.8, 88.7, and 96.3 mg), acetic acid (23.0, 30.3, and 33.5 mg), and NH_3_ (9.4, 11.5, and 12.0 mg). These results demonstrate that the incorporation of ACF increased the total amount of gas removed up to the 80% removal-efficiency threshold. However, because the addition of ACF also increased the total adsorbent mass, the higher cumulative uptake cannot be attributed solely to an increase in intrinsic adsorption capacity. Therefore, these results should be interpreted as improvements in the overall dynamic gas removal performance of the composite filters rather than enhancements in mass-normalized adsorption capacity. Considering material usage and filter thickness, the incorporation of a single ACF layer already provided a substantial increase in effective removal time and may therefore serve as a practical reference for the design of subsequent composite chemical filters. The non-proportional improvement in adsorption performance with increasing ACF layer number may be attributed to several factors. The incorporation of additional ACF layers increases the number of available adsorption sites and gas-contact interfaces, thereby extending the effective gas removal time. However, increasing the number of layers also increases the overall filter thickness, which may influence gas transport and the accessibility of adsorption sites within the multilayer structure. Under the investigated flow conditions, the additional adsorption sites provided by the second ACF layer may not be utilized as effectively as those introduced by the first layer. Furthermore, differences in local adsorption-site utilization and possible saturation behavior may contribute to the diminishing performance improvement. Nevertheless, these explanations remain hypothetical, as layer-specific pressure drop, internal mass-transfer resistance, and layer-specific adsorption saturation were not directly measured in the present study. To further evaluate the practical applicability of the filter developed in this study, its pressure-drop characteristics were compared with those of a commercial chemical filter under the same face-velocity conditions, as shown in Figure S3 in the Supplementary Materials. At face velocities of 2.0, 2.5, 3.0, and 3.3 m s^−1^, the developed filter exhibited pressure drops of 13.23, 17.64, 20.09, and 23.52 Pa, respectively, compared with 15.68, 19.11, 20.58, and 22.54 Pa for the commercial filter. Error bars represent the standard deviations (SD) of the pressure-drop measurements. These results indicate that the filter developed in this study exhibited comparable airflow resistance to the commercial reference under the investigated conditions. The filter had an areal weight of 800 g m^−2^. Although the developed and commercial filters were evaluated under identical pressure-drop testing conditions, differences in adsorbent loading, filter thickness, and structural composition may influence both adsorption performance and airflow resistance. Therefore, the comparison should be interpreted as an evaluation of overall filter-level performance rather than intrinsic adsorbent performance under identical structural conditions. Furthermore, environmental humidity, long-term operational stability, and possible particle shedding under continuous airflow conditions require further investigation to establish the practical applicability of the developed filter in semiconductor manufacturing environments. The dynamic gas removal experiments were conducted at laboratory test concentrations selected for comparative evaluation of the different adsorbents and filter configurations. These concentrations should not be considered representative of the low-level airborne molecular contaminant concentrations encountered in semiconductor cleanroom environments. Consequently, the observed t_80_ values cannot be directly extrapolated to filter service life under actual cleanroom operating conditions. Further testing at application-relevant pollutant concentrations, together with controlled humidity and long-term exposure conditions, is required to establish practical semiconductor manufacturing performance.

## 4. Conclusions

This study established a chemical-filter design strategy for enhancing the adsorption performance toward multiple types of gaseous pollutants through surface chemical modification of activated carbon, optimization of chemical-filter structure, and integration of activated carbon fiber (ACF) nonwoven layers. The results showed that different surface modification methods markedly altered the pore structure and surface functional groups of activated carbon. NH_4_OH-modified activated carbon exhibited a higher specific surface area and a more favorable microporous structure, accompanied in improved dynamic removal performance toward toluene, isopropyl alcohol (IPA), and acetic acid. In contrast, HNO_3_ activated carbon exhibited a lower specific surface area but a greater abundance of acidic and oxygen-containing surface functional groups, accompanied by improved NH_3_ removal performance. These observations suggest that surface chemical interactions may play an important role in NH_3_ adsorption. During chemical-filter fabrication, the adhesive content directly affected the degree of exposure of activated-carbon surface pores and, consequently, the adsorption performance. As the adhesive content increased, the activated-carbon surface became progressively covered, reducing the number of accessible adsorption sites. Among the adhesive-containing formulations investigated, 5 wt% adhesive resulted in the smallest reduction in dynamic gas removal performance. However, the long-term mechanical stability and particle retention of the filters were not quantitatively evaluated. The optimized chemical filters exhibited better adsorption performance toward toluene, IPA, acetic acid, and NH_3_ than the commercial chemical filter. The performance improvements for toluene and IPA were approximately 13.8% and 44.2%, respectively; the performance toward acetic acid was approximately twice that of the commercial filter, while NH_3_ performance was improved by approximately 88.5%. Further integration of ACF provided an additional enhancement in filter performance. The high micropore fraction of ACF suggests that the additional porous structure may have contributed to the observed improvement in dynamic gas removal performance. After the incorporation of one ACF layer, the adsorption performance toward toluene, IPA, acetic acid, and NH_3_ increased by approximately 10.5%, 14.7%, 31.6%, and 24%, respectively. In comparison, the additional improvement provided by a second ACF layer was relatively limited, suggesting that a single ACF layer may represent a practical configuration among those investigated, considering the observed performance improvement and the additional material required for a second layer. Overall, the main contribution of this study lies in integrating activated-carbon surface modification, adhesive-content optimization, and ACF nonwoven layers into a multilayer chemical-filter design, rather than developing a new carbon modification technique. These findings highlight the importance of considering both adsorbent properties and filter configuration for airborne molecular contaminant removal. The experimentally demonstrated findings include changes in surface characteristics, pore structure, dynamic gas removal performance, and pressure-drop behavior. In contrast, the proposed contributions of pore filling, intermolecular interactions, and acid–base interactions to gas adsorption are interpretations based on the characterization and adsorption results rather than directly verified mechanistic pathways. Further mechanistic investigations would be required to quantitatively distinguish these contributions. The pressure-drop measurements provide preliminary evidence of the airflow resistance characteristics of the developed composite filter, while additional evaluations of humidity effects, long-term stability, and particle shedding remain necessary to establish its practical applicability in semiconductor manufacturing environments.

## Figures and Tables

**Table 1:** Comparison of the time required for removal efficiency to decrease to 80% (t_80_) for commercial, pristine, and surface-modified activated carbons exposed to toluene, IPA, acetic acid, and NH_3_.

Sample	Toluene *t*_80_ (min)	IPA *t*_80_ (min)	Acetic Acid *t*_80_ (min)	NH_3_ *t*_80_ (min)
AC-CA	175	61	29	32
AC-Pure	144	44	16	1
AC-NH_4_OH	197	88	64	1
AC-HCOOH	150	75	28	16
AC-HNO_3_	76	53	19	57

**Table 2:** Comparison of the time required for removal efficiency to decrease to 80% (t_80_) for a commercial chemical filter and prepared chemical filters with varying adhesive contents and ACF layer numbers.

Sample ᵃ	Toluene *t*_80_ (min)	IPA *t*_80_ (min)	Acetic Acid *t*_80_ (min)	NH_3_ *t*_80_ (min)
CF-CA	167	52	19	26
CF-NH_4_OH-0%	197	88	64	57
CF-NH_4_OH-5%	191	75	38	50
CF-NH_4_OH-10%	158	48	31	44
CF-NH_4_OH-25%	71	43	14	39
CF-NH_4_OH-50%	5	26	3	33
CF-NH_4_OH-5%/ACF-1L	211	86	50	62
CF-NH_4_OH-5%/ACF-2L	220	93	55	65

ᵃ Sample designations containing CF-NH_4_OH refer to filters used for toluene, IPA, and acetic acid adsorption. For NH_3_ adsorption, the corresponding filters contained HNO_3_-modified activated carbon (CF-HNO_3_) with the same adhesive contents and ACF layer configurations.

## Data Availability

The data that has been used is confidential.

## Supplementary Materials

The following supporting information can be downloaded at https://www.mdpi.com/article/10.3390/polym18212774/s1, Figure S1: Effect of adhesive content on the characteristic removal time (t_80_) for toluene, IPA, NH_3_, and CH_3_COOH; Figure S2: N_2_ adsorption–desorption isotherm of pristine activated carbon fiber (ACF); Figure S3: Comparison of pressure drop as a function of face velocity between the filter developed in this study and a commercial chemical filter; Table S1: Textural properties of pristine and surface-modified activated carbons; Table S2: Cumulative dynamic adsorption capacities of selected surface-modified activated carbons up to the 80% removal-efficiency threshold (t_80_); Table S3: Textural properties of pristine activated carbon fiber (ACF); Table S4: Characteristic removal times (t_80_) and cumulative gas uptake up to t_80_ for composite filters containing different numbers of ACF layers.; Video S1: Demonstration of the continuous-flow gas adsorption testing system used to evaluate the gas removal performance of activated-carbon samples.
