# Supplementary material for: Facile Fabrication of Multilayer Chemical Filters Based on Modified Activated Carbon and Activated Carbon Fiber Nonwovens for Airborne Molecular Contaminant Removal in Semiconductor Manufacturing
Source: Polymers (Basel). 2026 Nov 6;18(21):2774. doi: 10.3390/polym18212774 (PMC13765957; doi:10.3390/polym18212774)
Supplement: Supplementary file 1 [file polymers-18-02774-s001.zip › Chiu-3 Supporting Information 30-Oct‐2026.pdf]

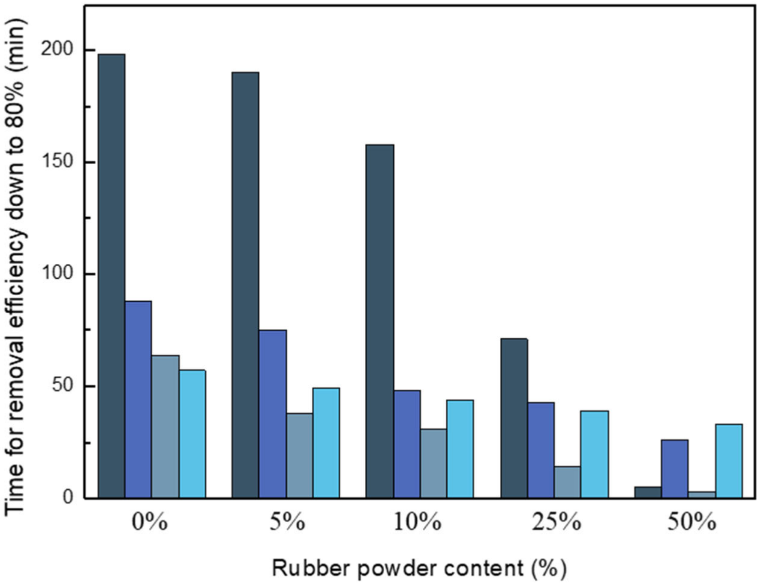

Figure S1. Effect of adhesive content on the characteristic removal time (t_{80}) for toluene, IPA, NH_3, and CH_3COOH.

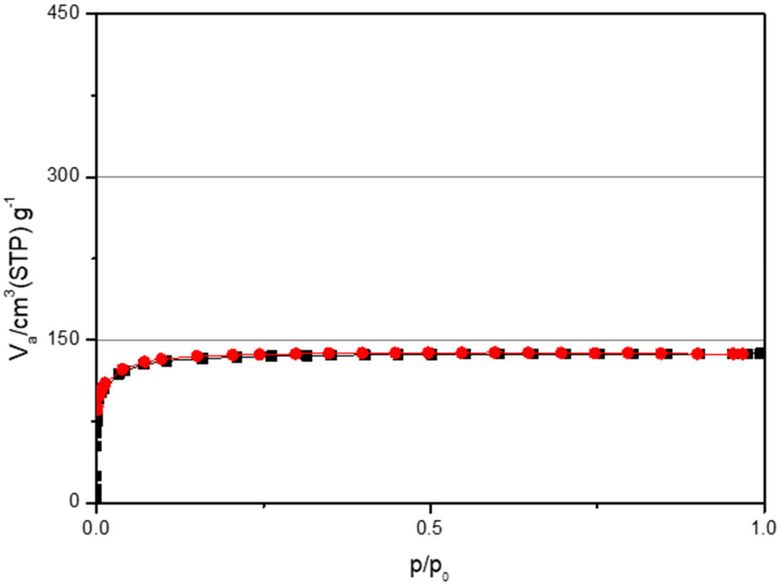

Figure S2. N_2 adsorption–desorption isotherm of pristine activated carbon fiber (ACF).

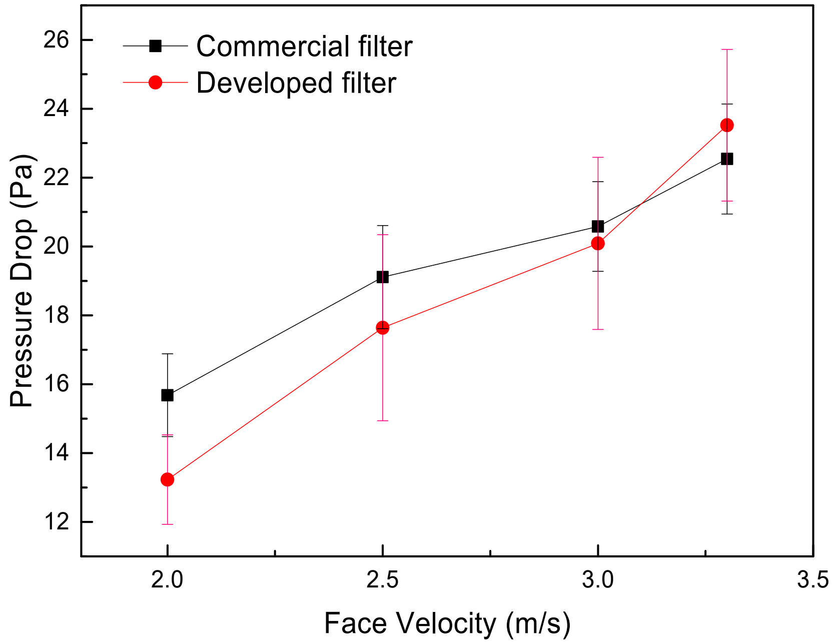

Figure S3. Comparison of pressure drop as a function of face velocity between the filter developed in this study and a commercial chemical filter. Error bars represent standard deviations (SD).

Table S1. Textural properties of pristine and surface-modified activated carbons.

Parameter	AC-Pure	AC-NH_4OH	AC-HCOOH	AC-HNO_3
BET specific surface area [a] ($m^2\ g^{-1}$)	1101 ± 7	1142 ± 10	1134 ± 8	1018 ± 11
Micropore volume ratio [b] (%)	94.53 ± 1.12	96.84 ± 2.35	95.36 ± 2.85	94.81 ± 2.54
Mesopore + macropore volume ratio [c] (%)	5.47 ± 1.12	3.16 ± 2.35	4.64 ± 2.85	5.19 ± 2.54
Micropore width [d] (nm)	0.8281 ± 0.0217	0.7972 ± 0.0285	0.8345 ± 0.0314	0.8326 ± 0.0252

[a] Specific surface area determined from the N_2 adsorption isotherm using the Brunauer–Emmett–Teller (BET) method.
[b] Fraction of the pore volume assigned to the micropore region in the pore-structure analysis.
[c] Fraction of the pore volume assigned to the non-micropore region; calculated as 100% minus the micropore volume fraction.
[d] Characteristic micropore width obtained from the pore-size analysis of the N_2 adsorption data.

Values are presented as mean ± standard deviation (SD) from three independent measurements (n = 3).

Table S2. Cumulative dynamic adsorption capacities of selected surface-modified activated carbons up to the 80% removal-efficiency threshold (t_{80}).

Gas	Sample	t_{80} (min)	q_{t80} (mg g^{-1})
Toluene	AC-NH_4OH	197	814.8
IPA	AC-NH_4OH	88	94.2
Acetic acid	AC-NH_4OH	64	39.4
NH_3	AC-HNO_3	57	11.0

Table S3. Textural properties of pristine activated carbon fiber (ACF).

Property	Mean ± SD (n = 3)
Surface area [a]	512.33 ± 39.01 m^2/g
Micropore volume ratio [b]	98.91 ±0.57 %

[a] Specific surface area determined from the N_2 adsorption isotherm using the Brunauer–Emmett–Teller (BET) method.
[b] Fraction of the pore volume assigned to the micropore region in the pore-structure analysis.

Table S4. Characteristic removal times (t_{80}) and cumulative gas uptake up to t_{80} for composite filters containing different numbers of ACF layers.

Gas	ACF layers	t_{80} (min)	Cumulative gas uptake (mg)
Toluene	0	191	753.0
Toluene	1	211	839.1
Toluene	2	220	876.4
IPA	0	75	77.8
IPA	1	86	88.7
IPA	2	93	96.3
NH_3	0	50	9.4
NH_3	1	62	11.5
NH_3	2	65	12.0
Acetic acid	0	38	23.0
Acetic acid	1	50	30.3
Acetic acid	2	55	33.5
